# Public health round-up

**DOI:** 10.2471/BLT.14.011014

**Published:** 2014-10-01

**Governments to discuss regulating e-cigarettes**

A WHO report – proposing regulatory action to limit the use of e-cigarettes – will be discussed at the Conference of the Parties (COP) to the Framework Convention on Tobacco Control in Moscow, the Russian Federation, from 13 to 18 October. The COP is the governing body of the international treaty and comprises 168 countries.

http://www.who.int/nmh/events/2014/backgrounder-e-cigarettes/en/

**Figure:**
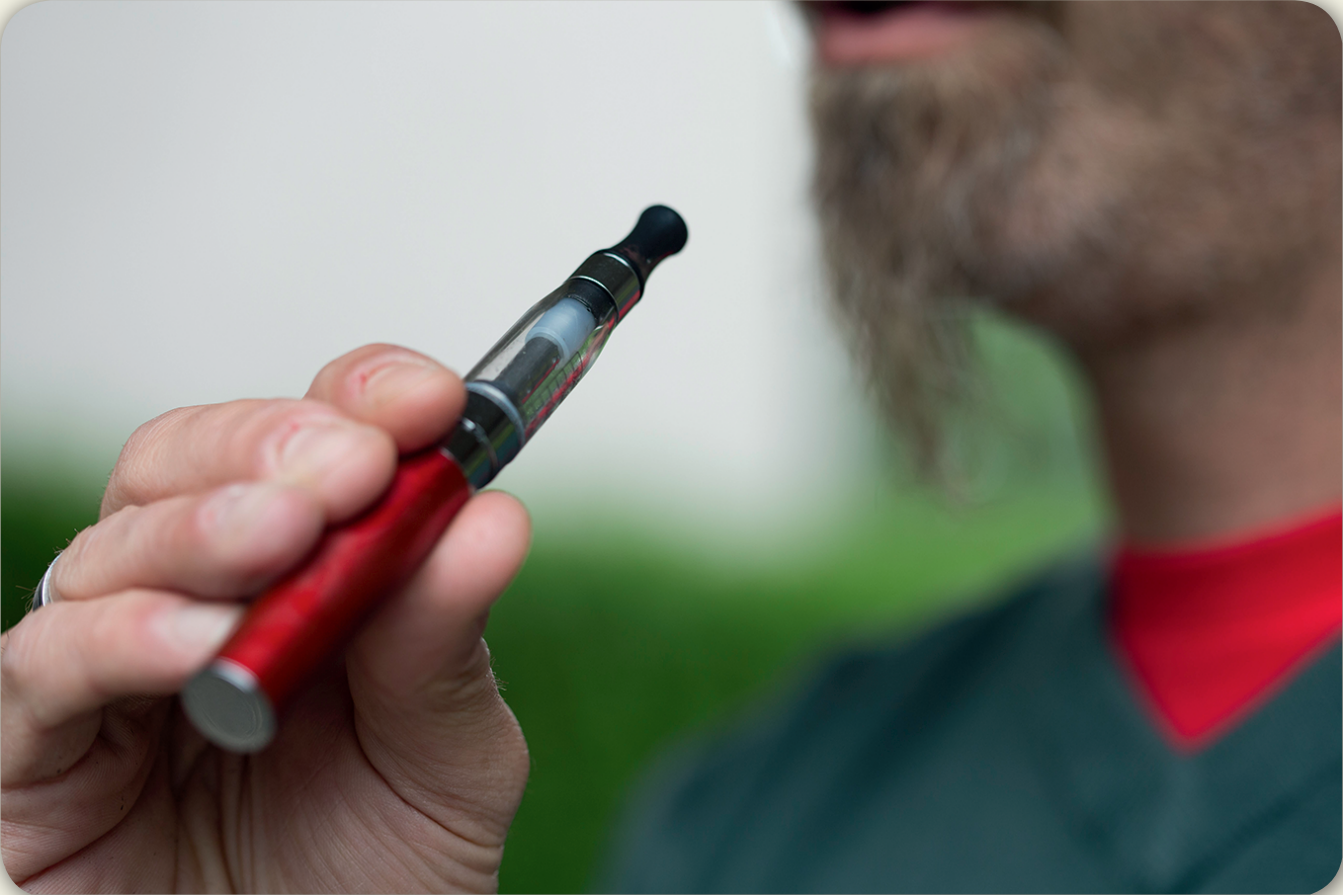
WHO

## New WHO Ebola plan 

The World Health Organization (WHO) issued a comprehensive plan to stop the Ebola virus disease outbreak in western Africa by June next year. The *Ebola response roadmap*, launched on 28 August, came as there was a leap in reported cases of 40% over three weeks and projections that capacity was needed to manage up to 20 000 more cases in the coming months.

“A massively scaled and coordinated international response is needed to support affected and at-risk countries in intensifying response activities and strengthening national capacities,” the roadmap says, calling for additional donor funding of US$ 490 million for the next six months alone.

The first cases in the current outbreak were reported in Guinea in March, though the outbreak has since been found to date back to December 2013. As of 12 September, 4784 cases had been reported in Guinea, Liberia and Sierra Leone, the three worst affected countries, and Nigeria and Senegal, which have suffered importations of the virus. With 2400 deaths, this is the largest Ebola virus disease outbreak on record.

The roadmap calls for the strengthening of laboratory capacity and the public health infrastructure in the affected countries, as well as reinforcements in other areas that are vital to the outbreak response, such as infection prevention and control training.

It aims to facilitate a huge scale-up in the supply of personal protective equipment, disinfectants and body bags to these countries.

The new plan builds on months of efforts to stem the outbreak. Government ministers from Guinea, Liberia, Sierra Leone and other African countries and the WHO Director-General, Dr Margaret Chan, called on the international community for more technical and financial support to stop the outbreak at a meeting from 2 to 3 July in Accra, Ghana.

At the end of July, an operations centre was established in Conakry, Guinea, to coordinate the outbreak response and the governments launched an initial *Ebola virus disease outbreak response plan* with WHO that outlined the actions that were needed, based on the situation at that time and an estimate of resource requirements. 

Since then the outbreak has spread to Nigeria and Senegal. The 28 August roadmap was informed by many WHO collaborators and partners, including health officials in the affected countries, the African Union, development banks, other United Nations agencies, Médecins Sans Frontières, US Centers for Disease Control and Prevention and countries providing direct financial support.

It is complemented by a separate United Nations-wide initiative that was launched on 17 September to address other areas such as the delivery of food and other provisions to the affected countries as well as water, sanitation and primary health care.

http://www.who.int/csr/disease/ebola

## Ukraine health emergency

The World Health Organization warned on 9 September that the crisis in eastern Ukraine was creating a “looming health emergency”. Hundreds of thousands of people have been displaced and were living “in varying and often exposed conditions as winter approaches, many without official status either as internally displaced persons or as refugees”. 

Since the conflict erupted earlier this year in the east European country, some 262 000 people have registered as internally displaced persons and more than 366 000 have fled as refugees to neighbouring countries, mainly the Russian Federation, as of 12 September, according to the United Nations High Commissioner for Human Rights.

At least 32 hospitals in eastern Ukraine have closed down, while 18 that have been shelled and damaged continued to offer limited care, according to the Ukrainian health ministry.

“The health needs for the people affected by the eastern Ukraine crisis are great and urgent action is needed to treat people who require all manner of care and services, from treatment for noncommunicable diseases and immunizations to the delivery of babies,” said Dr Dorit Nitzan, WHO Representative to the Ukraine.

WHO and its partners are responding by providing medical supplies and setting up emergency primary health-care mobile units for the displaced. In collaboration with the Ukrainian authorities, WHO is helping to step up the national early warning system for infectious disease outbreak prevention and control. A vaccination campaign for polio and measles is also planned with UNICEF [the United Nations Children’s Fund].

In mid-August, WHO appealed to donors for US$ 14 million to respond to the health emergency in Ukraine.

## Donation for Iraq crisis

The World Health Organization received US$ 49 million from Saudi Arabia – a much needed boost for its work responding to the health needs of millions of people affected by the crisis in Iraq. This donation is believed to be the largest ever contribution WHO has received for a specific humanitarian crisis.

A recent upsurge in violence in June resulted in massive population movement that has placed additional strain on the health system, which is already struggling to address the needs of more than 250 000 Syrian refugees in northern Iraq.

The donation from Saudi Arabia enables WHO and its partners to scale up its response to disease outbreaks, malnutrition, medicine shortages and overburdened hospitals and clinics, according to Dr Syed Jaffar Hussain, WHO’s Representative to Iraq.

It will also help WHO and its partners ensure the provision of health services, including emergency obstetric care for expectant mothers, noncommunicable diseases care and psychosocial support to many more people.

“Humanitarian health-care providers will tackle medical complications resulting from malnutrition by providing targeted support to 350 000 people, including children under five, pregnant and lactating women and patients with severe malnutrition,” said Hussain.

Last month, WHO announced that despite the ongoing conflict and the resulting violence and displacement across Iraq, a mass polio immunization campaign had reached 3.75 million of 4 million children under the age of five in 13 high-risk governorates.

The five-day campaign between 10 and 14 August was organized by the Iraqi ministry of health with the support of UNICEF and WHO. Confirmation of two cases of polio in Iraq in February and April of this year ended a 14-year period during which Iraq had been polio free.

http://www.who.int/hac/crises/irq/releases/saudi_arabia_support

**Cover photo**

Communities affected by the outbreak of Ebola virus disease in western Africa need reliable and credible information about the disease to protect themselves and prevent further infections. This month’s cover shows a campaign poster being put up in Sierra Leone.

**Figure:**
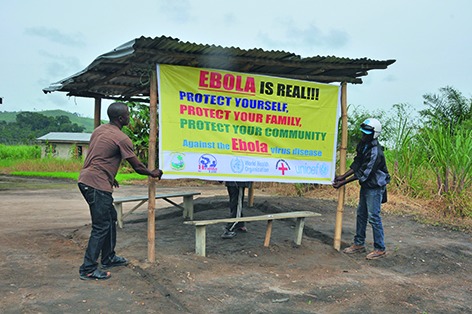
WHO

## Prison health conference

A conference devoted to promoting health services in the criminal justice system takes place this month in Portlaoise, Ireland. This year’s theme is prisoner empowerment and improvement of their lives in the community once they are released.

The conference, from 1 to 3 October, is expected to gather health and criminal justice specialists from about 18 countries. It is organized by WHO’s European Regional Office in collaboration with the Irish Prison Service and Public Health England. The WHO Health in Prison Programme started in 1995 with the aim of promoting health in the criminal justice system, as part of the overall public health agenda. 

http://www.euro.who.int/en/media-centre/events/events/2014/10/international-conference-on-prison-health

## People-centred health care 

A new WHO strategy places people at the centre of health services and calls for a radical new approach to funding, managing and delivering health services.

The draft text will be presented at the Third Global Symposium on Health Systems Research, from 30 September to 3 October in Cape Town, South Africa, attended by researchers, policy-makers and other members of the global public health community.

The draft strategy on people-centred health services is important in the context of countries moving towards or trying to maintain universal coverage of health services.

“People-centred health services is an innovative approach that adopts individuals’, families’, and communities’ perspectives as participants and beneficiaries of trusted health systems that respond to their needs and preferences,” says Dr Edward Kelley, director of WHO’s Department of Service Delivery and Safety.

http://www.who.int/topics/health_systems

## New health systems journal

A new open-access journal aims to give authors from developing countries an opportunity to share their research findings on building and strengthening health systems with a wide audience.

*Strengthening health systems* was launched in July. Unlike most open-access journals, it does not charge author fees. The journal was conceived by the Foundation for Professional Development, which is based in South Africa, and is funded by the United States Agency for International Development (USAID).

www.shsjournal.org

**Looking ahead**

**19–21 November 2014** – **Food and Agriculture Organization/WHO Second International Conference on Nutrition (ICN2)** at FAO Headquarters, Rome, Italy. http://www.fao.org/about/meetings/icn2
**1 December** – **World AIDS Day**. http://www.worldaidsday.org
**3–6 December** – **World Cancer Congress** in Melbourne, Australia. http://www.worldcancercongress.org
**26 January–3 February** **2015** – **WHO Executive Board meeting** in Geneva, Switzerland. http://apps.who.int/gb/e/e_eb136.html

